# Short- and long-term effects of an electronic medication management system on paediatric prescribing errors

**DOI:** 10.1038/s41746-022-00739-x

**Published:** 2022-12-13

**Authors:** Johanna I. Westbrook, Ling Li, Magdalena Z. Raban, Virginia Mumford, Tim Badgery-Parker, Peter Gates, Erin Fitzpatrick, Alison Merchant, Amanda Woods, Melissa Baysari, Cheryl McCullagh, Ric Day, Madlen Gazarian, Michael Dickinson, Karla Seaman, Lucciano Dalla-Pozza, Geoffrey Ambler, Peter Barclay, Alan Gardo, Tracey O’Brien, Draga Barbaric, Les White

**Affiliations:** 1 Australian Institute of Health Innovation, Macquarie University, Sydney, Australia; 2 Faculty of Medicine and Health, The University of Sydney, Sydney, Australia; 3 Sydney Children’s Hospitals Network, Sydney, Australia; 4 Faculty of Medicine and Health, University of New South Wales, Sydney, Australia; 5 Cancer Institute NSW, Sydney, Australia

**Keywords:** Paediatrics, Risk factors

## Abstract

Electronic medication management (eMM) systems are designed to improve safety, but there is little evidence of their effectiveness in paediatrics. This study assesses the short-term (first 70 days of eMM use) and long-term (one-year) effectiveness of an eMM system to reduce prescribing errors, and their potential and actual harm. We use a stepped-wedge cluster randomised controlled trial (SWCRCT) at a paediatric referral hospital, with eight clusters randomised for eMM implementation. We assess long-term effects from an additional random sample of medication orders one-year post-eMM. In the SWCRCT, errors that are potential adverse drug events (ADEs) are assessed for actual harm. The study comprises 35,260 medication orders for 4821 patients. Results show no significant change in overall prescribing error rates in the first 70 days of eMM use (incident rate ratio [IRR] 1.05 [95%CI 0.92–1.21], but a 62% increase (IRR 1.62 [95%CI 1.28–2.04]) in potential ADEs suggesting immediate risks to safety. One-year post-eMM, errors decline by 36% (IRR 0.64 [95%CI 0.56–0.72]) and high-risk medication errors decrease by 33% (IRR 0.67 [95%CI 0.51–0.88]) compared to pre-eMM. In all periods, dose error rates are more than double that of other error types. Few errors are associated with actual harm, but 71% [95%CI 50–86%] of patients with harm experienced a dose error. In the short-term, eMM implementation shows no improvement in error rates, and an increase in some errors. A year after eMM error rates significantly decline suggesting long-term benefits. eMM optimisation should focus on reducing dose errors due to their high frequency and capacity to cause harm.

## Introduction

Medication errors are a recognised persistent problem in paediatrics^1^. Information technologies including electronic medication management systems (eMM, also known as e-prescribing or Computerised Provider Order Entry [CPOE]) have been advanced to reduce medication errors in this population^2–4^. Multiple studies of eMM use and its effects in adult hospitals have been conducted^5^, with a recent systematic review finding nine of 10 studies reported a significant reduction in prescribing error rates^5^. However, reviews on this topic persistently report marked inconsistencies in study quality, error definitions and denominators^5–7^. Few studies have investigated the effects of this technology in paediatric hospitals^1^.

Prescribing in children is complex as decisions often require information about weight, age, dosing ranges and off-label use of medicines, and this increases the risk of medication errors^8,9^. The consequences of prescribing errors can be significant given children’s physiological and developmental vulnerabilities^10^. Concerns have been raised that eMM systems have often not been well-adapted to paediatric medication challenges^11–14^ (e.g., the increased complexity of weight-based dose calculations in children and the absence of detailed paediatric-specific medicines information in the decision support provided). Hence findings from eMM studies in adult hospitals may not be directly extrapolated to paediatric populations.

eMM systems have multiple mechanisms by which they are designed to support safe prescribing and reduce errors. These include: improved legibility of medication orders; structured order sentences for selection and editing; decision support (both passive e.g. online information resources and active e.g. alerts) available at the point of prescribing.

eMM evaluation studies undertaken in paediatrics have largely employed before and after designs, and most have focused on systems used in intensive care^5,9,15^. The existing limited evidence suggests that eMM may be more effective in reducing only certain error types in paediatrics. For example, Walsh et al.^16^ found paediatric eMM systems were effective in reducing incomplete/wrong order errors but not dose errors, and a review by Tolley et al.^17^ identified eMM features which contributed to dose errors in paediatrics. Greater knowledge of these outcomes is needed to inform system design and use. No randomised controlled trials of the effectiveness of eMM to reduce medication errors on general paediatric wards have been conducted, and few studies have attempted to quantify changes in actual harm associated with eMM use^1,5,18^.

As the adoption of clinical information systems continues worldwide, questions about how best to implement these systems in safe and effective ways remain. The strengths and weaknesses of sequential versus whole of organisation (big bang) roll-outs are advocated with limited evidence to guide decision-making^19,20^. Feasibility and resource issues will often drive the implementation strategy. eMM evaluation studies have focused on measurement at specific points in time and have provided limited evidence of the temporal impact of systems on medication safety^5^. Such information would be valuable in identifying both when implementation support is most required and at what point medication safety benefits may be expected.

In this study, we conduct a stepped-wedge cluster randomised controlled trial (SWCRCT) with an additional one-year follow-up to assess the short- and long-term effectiveness of an eMM system to reduce prescribing errors, their potential severity and associated actual harm at a major paediatric referral hospital. We demonstrate no initial benefit from eMM in the first 70-days of use, with increases in some error types posing potentially greater patient risk during this period. Prescribing error rates one-year after eMM significantly decline from pre-eMM rates. Dose errors are the most frequent error type and are most often associated with patient harm. Our findings indicate the need for close attention to support staff in system training and integration of use into clinical work practices, along with monitoring of errors during the first months of system use. Optimisation of paediatric eMM systems should particularly focus on features to reduce dose errors and their effects.

## Results

### Characteristics of study periods

In total 35,260 medication orders for 4821 patients across all study periods (4509 unique patients) were retrospectively reviewed for errors. Table 1 reports the characteristics of patients and orders.

**Table 1:** Characteristics of patients and medication orders in the three periods.

	Control period	Intervention period	
	Paper	First 70 days of eMM	One-year post-eMM
Number of patients	1686	2096	1039
Number (%) female	691 (40.98)	838 (39.98)	460 (44.27)
Mean (SD) age, months	73.9 (62.4)	88.4 (63.4)	86.1 (63.5)
Median (IQR) age, months	55.5 (18.0–124.8)	85.0 (27.0–142.0)	80.0 (26.0–139.0)
Number of admissions	1769	2298	1201
Number of patient-days	5252	8384	4458
Number of orders	9635	16,734	8891
Number (%) of orders for high-risk medications (see Box 1)	1200 (12.45)	2274 (13.59)	1334 (15.00)

### Changes in prescribing error rates in the short- and long-term post-eMM

Overall, clinical prescribing error rates did not significantly change (IRR 1.05) in the short-term (first 70 days of eMM use), but there was a significant 62% increase (IRR 1.62) in potential ADEs (Table 2). Procedural prescribing errors significantly declined (IRR 0.65) in the short-term (Table 2 and further details available in Supplementary Tables 1 and 2).

**Table 2:** Prescribing crude error rates and adjusted incidence rate ratios (IRRs) for the SWCRCT (short-term impact of eMM).

	Control (Paper)	Intervention (first 70 days of eMM)	IRR (95% CI)^a^ (eMM vs Paper)	*P* value^a^
All clinical prescribing errors			–	
Number of patients	1686	2096		
Number of medication orders	9635	16,734		
Number of clinical prescribing errors	1399	2942		
Error rate^b^ per 100 orders (95% CI)	14.52 (13.78–15.30)	17.58 (16.96–18.23)	1.05 (0.92–1.21)	0.45
Potential ADEs (clinical prescribing errors with a potential severity score ≥3)				
Number of potential ADEs	353	812		
Error rate^b^ per 100 orders (95% CI)	3.66 (3.30–4.07)	4.85 (4.53–5.20)	1.62 (1.28–2.04)	<0.001
Clinical prescribing errors involving high-risk medications				
Number of patients with high-risk medication orders	799	1228		
Number of high-risk medication orders	1200	2274		
Number of clinical prescribing errors involving high-risk medications	232	390		
Error rate^b^ per 100 high-risk medication orders (95% CI)	19.33 (17.00–21.99)	17.15 (15.53–18.94)	0.88 (0.66–1.16)	0.35
All procedural errors^c^				
Number of patients with procedural errors reviewed	1162	1429		
Number of medication orders	6698	11,441		
Number of procedural errors	7652	7699		
Procedural error rate^b^ per 100 orders (95% CI)	114.24 (111.71–116.83)	67.29 (65.81–68.81)	0.65 (0.61–0.70)	<0.001

^a^Adjusted incidence rate ratio (IRR), 95% CIs and *P* values from generalised linear mixed models adjusting for patient age, study time, and clustering by ward (unit of randomisation) and patient. ADE is adverse drug event.

^b^Crude error rates and 95% CIs are from simple Poisson models.

^c^Procedural errors were assessed from a random sample comprising 68.5% of patients during the entire SWCRCT period. Details of changes in procedural errors by error type are in Supplementary Tables 1 and 2.

One-year post-eMM, clinical prescribing error rates had significantly declined by 36% (IRR 0.64) compared to when paper medication charts (control) were used (Table 3). There was also a 33% reduction in errors associated with high-risk medications between the control and one-year post-eMM (IRR 0.67) (Table 3), and potential ADEs returned to a similar level to the control period.

**Table 3:** Prescribing error rates and adjusted incidence rate ratios (IRRs) comparing control and one-year post-eMM periods.

	Control (Paper)	Intervention (one-year post-eMM)	IRR (95% CI)^a^ (one-year post-eMM vs Paper)	*P* value^a^
All clinical prescribing errors			–	
Number of patients	1686	1039		
Number of medication orders	9635	8891		
Number of clinical prescribing errors	1399	1043		
Error rate^b^ per 100 orders (95% CI)	14.52 (13.78–15.30)	11.73 (11.04–12.46)	0.64 (0.56–0.72)	<0.001
Potential ADEs (clinical prescribing errors with a potential severity score ≥3)				
Number of potential ADEs	353	299		
Error rate^b^ per 100 orders (95% CI)	3.66 (3.30–4.07)	3.36 (3.00–3.77)	0.93 (0.72–1.19)	0.55
Clinical prescribing errors involving high-risk medications				
Number of patients with high-risk medication orders	799	607		
Number of high-risk medication orders	1200	1334		
Number of clinical prescribing errors involving high-risk medications	232	176		
Error rate^b^ per 100 high-risk medication orders (95% CI)	19.33 (17.00–21.99)	13.19 (11.38–15.29)	0.67 (0.51–0.88)	0.004

^a^Adjusted incidence rate ratio (IRR), 95% CIs and *P* values from generalised linear mixed models adjusting for patient age, study time, and clustering by ward (unit of randomisation) and patient. ADE is adverse drug event.

^b^Crude error rates and 95% CIs are from simple Poisson models.

### Changes in types of clinical prescribing errors in the short- and long-term post-eMM

The most frequent types of clinical prescribing errors in all periods were dose, route, frequency and duplicate drug therapy errors, in total making up over 90% of errors in each period (Table 4). Dose errors comprised more than 40% of all clinical prescribing errors in all periods. Overdose error rates were consistently higher in all periods compared to underdose errors (Table 4).

**Table 4:** Clinical prescribing error rates by type and changes over study periods.

Error type	Control—Paper (*n* = 9635 orders)		Intervention—First 70 days of eMM (*n* = 16,734 orders)			Intervention—One-year post-eMM (*n* = 8891 orders)		
	No. Errors	Errors/100 orders (95% CI)^a^	No. Errors	Errors/100 orders (95% CI)^a^	IRR^b^ eMM vs Paper	No. Errors	Errors/100 orders (95% CI)^a^	IRR^b^ One-year post-eMM vs Paper
Wrong dose	644	6.68 (6.19–7.22)	1288	7.70 (7.29–8.13)	1.22 (1.04–1.43)	534	6.01 (5.52–6.54)	0.79 (0.68–0.93)
Overdose	308	3.20 (2.86–3.57)	674	4.03 (3.74–4.34)	1.22 (0.96–1.55)	247	2.78 (2.45–3.15)	0.79 (0.63–1.01)
Underdose	223	2.31 (2.03–2.64)	408	2.44 (2.21–2.69)	1.25 (0.95–1.66)	183	2.06 (1.78–2.38)	0.83 (0.64–1.07)
Wrong frequency	315	3.27 (2.93–3.65)	522	3.12 (2.86–3.40)	0.92 (0.73–1.16)	157	1.77 (1.51–2.06)	0.47 (0.36–0.61)
Wrong route	199	2.07 (1.80–2.37)	396	2.37 (2.14–2.61)	1.65 (1.20–2.27)	77	0.87 (0.69–1.08)	0.44 (0.27–0.71)
Duplicated drug therapy	140	1.45 (1.23–1.71)	457	2.73 (2.49–2.99)	1.82 (1.26–2.63)	205	2.31 (2.01–2.64)	1.48 (1.05–2.09)
Wrong drug	24	0.25 (0.17–0.37)	62	0.37 (0.29–0.48)	1.82 (0.66–5.00)	15	0.17 (0.10–0.28)	0.49 (0.11–2.23)
Drug–drug interaction	20	0.21 (0.13–0.32)	71	0.42 (0.34–0.54)	0.69 (0.23–2.09)	17	0.19 (0.12–0.31)	0.39 (0.09–1.73)
Allergy	20	0.21 (0.13–0.32)	19	0.11 (0.07–0.18)	0.45 (0.12–1.75)	13	0.15 (0.08–0.25)	3.17 (0.48–20.9)
Inadequate monitoring	19	0.20 (0.13–0.31)	40	0.24 (0.18–0.33)	1.19 (0.34–4.19)	5	0.06 (0.02–0.14)	–

– indicates too few errors for modelling (Supplementary Table 3 reports remaining error categories).

^a^Crude error rates and 95% CIs are from simple Poisson models.

^b^Adjusted incidence rate ratio (IRR) and 95% CIs are from generalised linear mixed models adjusting for study time (immediate post-eMM model only), patient age, and clustering by ward and patient.

In the short-term, rates for three error types increased: wrong dose (IRR 1.22), route (IRR 1.65) and duplicate therapy (IRR 1.82) errors (Table 4) relative to the control period. Wrong frequency errors remained relatively constant during the first 70 days of eMM use.

One-year post-eMM, wrong dose (IRR 0.79), wrong route errors (IRR 0.44) and wrong frequency errors (IRR 0.47) had declined compared to the control period. However, duplicate drug therapy error rates continued to be higher than when paper medication charts (control) were used (IRR 1.48) (Table 4).

### Potential ADEs and evidence of error detection by staff

In total 27.2% (1464/5384) of clinical prescribing errors were rated with a potential harm score of ≥3. There was little change in this proportion over the study periods (Table 5).

**Table 5:** Severity of potential harm from clinical prescribing errors by study period.

Potential harm^a^ severity	Control—Paper		Intervention—First 70 days of eMM		Intervention—One-year post-eMM	
	*n*	% (95% CI)	*n*	% (95% CI)	*n*	% (95% CI)
1: No/minimal harm	382	27.3% (24.5–30.1)	797	27.1% (25.2–29.1)	281	26.9% (23.7–30.3)
2: Temporary harm requiring monitoring	664	47.5% (44.7–50.3)	1333	45.3% (43.4–47.3)	463	44.4% (41.1–47.7)
3: Temporary harm requiring intervention	299	21.4% (18.6–24.2)	686	23.3% (21.4–25.3)	266	25.5% (22.2–28.8)
4: Permanent harm requiring intervention	48	3.4% (0.6–6.3)	112	3.8% (1.9–5.8)	28	2.7% (0–6.0)
5: Potential death	6	0.4% (0–3.3)	14	0.5% (0.0–2.5)	5	0.5% (0.0–3.8)
Total of prescribing errors with a score ≥3	353	25.2% (23.0–27.6)	812	27.6% (26.0–29.3)	299	28.7% (25.9–31.5)

^a^Definitions provided in Supplementary Table 6. Confidence intervals (CIs) for the potential harm categories are 95% simultaneous multinomial CIs. For the total, CIs are exact (Clopper–Pearson) binomial 95% CIs.

A small proportion of clinical prescribing errors was detected by staff, but this proportion increased significantly with eMM implementation (test of equal proportions *χ*^2^ = 29.6, 1 df, *p* < 0.001). In the control period, 9.3% (95% CI 7.8–10.9) of errors had some evidence of detection. In the short-term, this increased to 15.4% (95% CI 14.1–16.7) and at one-year post-eMM was 17.3% (95% CI 15.0–19.7).

### Actual harm associated with clinical prescribing errors in the SWCRCT

In total, 368 errors involving 172 patients were reviewed by the harm assessment panels. For 21 (12.2%) patients with 40 clinical prescribing errors, there was evidence in patients’ records of associated harm. Of these 21 patients, 8 (18 errors) experienced errors resulting in minor harm, 12 (21 errors) moderate and 1 serious harm (1 error). The harm panel rated the likelihood that the harm was caused by the error as possible in 9 cases, probable in 8 cases, and certain in 4 cases. Dose errors were attributed to the actual harm occurring in 71% (15/21; 95% CI 50–86) of patients experiencing harm (control period 8 dose errors/11 patients with harm; first 70 days post-eMM 7 dose errors/10 patients) (Supplementary Table 4). Table 6 reports prescribing errors associated with actual harm by a range of denominators.

**Table 6:** Clinical prescribing errors associated with actual harm (actual ADEs) for control and short-term intervention periods.

Prescribing errors with actual harm	Control—Paper (number of errors with actual harm = 14)	Intervention—First 70 days of eMM (number of errors with actual harm = 26)
per 1000 orders	1.45 [95% CI 0.87–2.44]	1.55 [95% CI 1.06–2.28]
per 1000 errors	10.01 [95% CI 5.97–16.73]	8.84 [95% CI 6.04–12.92]
per 1000 patient-days	2.66 [95% CI 1.59–4.47]	3.10 [95% CI 2.12–4.54]
per 100 admissions	0.79 [95% CI 0.47–1.32]	1.13 [95% CI 0.77–1.65]

Denominator numbers are presented in Table 1.

*eMM* electronic medication management system, *CI* confidence interval (binomial Wilson method).

## Discussion

We aimed to assess the effectiveness of eMM use in paediatrics to reduce prescribing errors both in the short- and long-term. We found that in the short-term there was no significant decrease in overall clinical prescribing error rates (IRR 1.05; 95% CI 0.92–1.21). However, potential ADEs increased by 62% (IRR 1.62; 95% CI 1.28–2.04).

Our one-year follow-up showed a significant 36% reduction (IRR 0.64; 95% CI 0.56–0.72) in overall clinical prescribing errors compared to the paper period. Prescribing errors involving high-risk medications decreased by 33% (IRR 0.67; 95% CI 0.51–0.88) and potential ADEs returned to a rate similar to the control (IRR 0.93; 95% CI 0.72–1.19).

Very limited comparative data are available, with differences in study designs and error definitions and identification processes applied^2,5^. To date, decision makers have largely relied upon evidence of eMM effectiveness in paediatrics from two studies conducted on general paediatric wards in the United States undertaken between 2006 and 2008^16,21^. One^21,22^ uncontrolled pre/post study showed a reduction in medication errors post-eMM and the second^16^ (an interrupted time series study) no change. A further small uncontrolled pre-post 2006 UK study^23^ reported an increase in prescribing errors 6 months post-eMM.

The time periods over which previous studies have examined effectiveness have also varied. Studies by Barber et al.^23^ and Walsh et al.^16^ reported no reduction in medication error rates at 6 and 9 months post-eMM, while Holdsworth et al.^21^ found significant reductions at around 15 months post-eMM. While these studies provide a small comparative evidence-base, together with our findings they are broadly consistent in demonstrating that beneficial effects of eMM on prescribing may not be apparent for a period of around 12 months. This provides a useful time parameter to inform future studies regarding when post-implementation assessments should be considered and when benefits may be likely to commence.

To understand the experiences of clinical staff during the implementation and early stages of eMM use, in parallel with the SWCRCT, we conducted 122 short interviews with doctors and nurses on all wards 1, 3, 6 and 26 weeks post-eMM^24^. Inexperience with system use and issues with system usability, functionality and accessibility were frequently reported in the immediate weeks following implementation, with clinicians suggesting that these factors contributed to delayed medication delivery and reduced medication safety^24^. Dealing with hybrid paper and computer medication systems was also identified as problematic. These themes were generic across wards during the early weeks of system use and are factors which assist in explaining the failure of prescribing errors to decrease in the immediate post-eMM period.

Interview findings demonstrated that issues raised by staff changed over time^24^. Six months post-eMM, many initial problems reported by staff (e.g. inexperience with use) were no longer mentioned. At 6-months post-eMM new themes emerged with clinicians increasingly reporting that the system was making a positive contribution to medication safety, consistent with the changes in error rates observed longer term. However, new issues also became apparent, including concerns about new types of errors, e.g. drop-down menu selection errors, associated with system use^17,25^. Some of these technology-related errors may decrease as users become more familiar with the system and identify ways to work around system limitations. Thus, a reduction in these technology-related errors following increased eMM system familiarity may also have contributed to both the lack of change in prescribing error rates in the short-term and the later observed reduction in error rates one-year post-eMM.

An important new finding was evidence of an increase in some categories of high-volume clinical prescribing errors during the first 70 days of eMM use. Four error types accounted for over 90% of all errors in each study period, and rates for three of these common error types increased in the short-term. These results suggest the eMM system had generalised effects on prescribing, consistent with users’ initial unfamiliarity and inexperience with system use, and broad disruptions to workflows^26^, all of which were reported by users in the early weeks of system use^24^.

Training was mandatory for staff in order to use the system and was provided close to the time of implementation. Yet many still reported considerable challenges in system use. This situation is likely a reflection of the differences between using a system in a training situation compared to a real-world setting where contextual factors such as time pressures, interruptions and integration with other related work practices come into operation. It is very difficult to mimic these real-world work demands and pressures within a system training environment. These findings re-emphasise the importance of high levels of support, training and close monitoring during the first several months of use.

One-year post-eMM, reductions in three of the four common paediatric prescribing errors had occurred. These reductions suggest that, in the longer term, with users’ increased familiarity with the system, the core functionality of eMM (e.g. the ability to select from standardised order sentences, legibility of all orders, decision support such as dose guidance) contributed to reduced prescribing errors. Duplicate drug therapy errors increased in the short-term post-eMM and remained higher one-year post-eMM. A doubling of duplicate therapy errors was identified in a recent before and after study of eMM in a paediatric intensive care unit in Ireland^27^. Feedback from our study pharmacists indicated a likely contributing factor was that clinicians seeking to correct an order in the eMM would type a new order but then fail to cease the original order, leading to duplicate orders being active. Hybrid paper-eMM situations also contributed to duplicate therapy errors, with some orders appearing both on paper and electronic charts during the implementation period. Some medications remained on separate paper charts, for example sliding scale insulin orders and some specialised pain medications, and these are likely to have contributed to duplicate drug therapy errors in the longer term. These error types should be amenable to improvement by reducing hybrid systems, alerting users via training, and system design enhancements.

Wrong dose errors are the most concerning type of error, given their high frequency and potential for harm. Dose errors, including 10-fold dose errors^27,28^, have consistently been identified as problematic in paediatrics, despite the introduction of eMM^9,14,16,27,28^. Reasons include the complexity of dose calculations for paediatrics and the sometimes limited guidance available to clinicians regarding medication use in children with specific conditions.

These findings indicate that a key focus for optimisation of eMM should be reducing dose errors. System features which provide support and guidance for prescribers, such as indication-dose alerts, weight-based filtering of order sentences, alerts to indicate major variations in patient weight that may result in dosing errors, and dose-weight calculators should be considered^17,29^. Such efforts to reduce dose errors are likely to bring the most benefit to reducing the risk of patient harm, in tandem with education in safe paediatric prescribing principles^8,30^. Further investigation of the mechanisms by which prescribing errors occur when an eMM is used can be particularly helpful in identifying design feature changes or tips for users in order to avoid common errors. To share this knowledge more widely as it becomes available, an outcome from this study is the publication of the Health Innovation Series on eMedication Safety^31^ which provides one-page evidence-based recommendations for system optimisation and user tips.

Overall, we found a low rate of prescribing errors associated with actual harm. Harm rates in the control and intervention periods were similar. However, among patients with errors resulting in actual harm, most cases (71%) involved dose errors (both with paper prescribing 73% and in the first 70 days post-eMM 70%).

Few studies have assessed actual harm associated with medication errors. Holdsworth et al.^21^ compared harm associated with multiple types of medication errors (including administration errors) occurring in paediatric wards and intensive care units. They reported a change in errors causing harm from 3.8/100 admissions to 2.2/100 post-eMM, considerably higher than found in our study (0.79/100 admissions pre and 1.13 post). A limitation of our study was that our harm identification relied upon retrospective record reviews. In contrast, Holdsworth et al.^21^ conducted prospective record reviews and interviews with staff close in time to when errors occurred. This enabled the capture of information which may never be recorded in patients’ records. Thus, our rates of prescribing errors causing actual harm are likely to be an underestimate of the true rate.

For over 80% of all clinical prescribing errors identified in our retrospective record audits there was no evidence that errors had been detected by staff. Thus, specific actions, such as monitoring or testing for signs or symptoms indicative of adverse effects, rarely occurred. An absence of investigations and monitoring information, which might provide clues as to possible adverse effects, limited the capacity of our panels to identify harm. An absence of documented evidence was generally equated to be an absence of adverse effects by our clinical harm panels. The clinical complexity of cases reviewed was also noted to be generally very high and abstracting the potential consequences of an error for children with complicated clinical presentations who were on a range of medications was often difficult for the panel. These challenges should be considered by future researchers embarking upon this process. Due to resource constraints, we did not conduct harm panels one-year post-eMM and so cannot determine whether errors associated with harm decreased in the long-term.

The SWCRCT design with one-year follow-up allowed a rigorous evaluation of the effectiveness of the eMM system to reduce prescribing errors. The 12-month follow-up study was an uncontrolled pre-post assessment. The research team had close and ongoing consultation with the hospital during the entire study and no significant medication safety interventions were instituted during this time period. However, we cannot rule out other factors that may have contributed to the reduction in error rates seen at the one-year follow-up. The study was limited to a specialist paediatric hospital, and so generalisability to other sites is unclear. Retrospective prescribing error harm assessments were limited by the level and quality of medical record documentation.

Our findings demonstrated no initial benefit from eMM in the first 70-days of use and suggest measurable risks to patient safety in this period due to increases in potentially harmful prescribing errors. Thus, close attention to support staff in training and integration of system use into clinical work practices, along with monitoring of errors during the first few months of system use should be a priority. One year later, prescribing error rates had significantly declined. Future optimisation of paediatric eMM systems should focus on features to reduce dose errors due to their high frequency and greater capacity to cause harm in this population.

## Methods

### Study design and setting

A SWCRCT with an additional one-year follow-up was undertaken at a 310-bed major urban paediatric tertiary referral hospital, one of two such hospitals serving the 8.2 million population of New South Wales, Australia^32^.

**Fig. 1:**
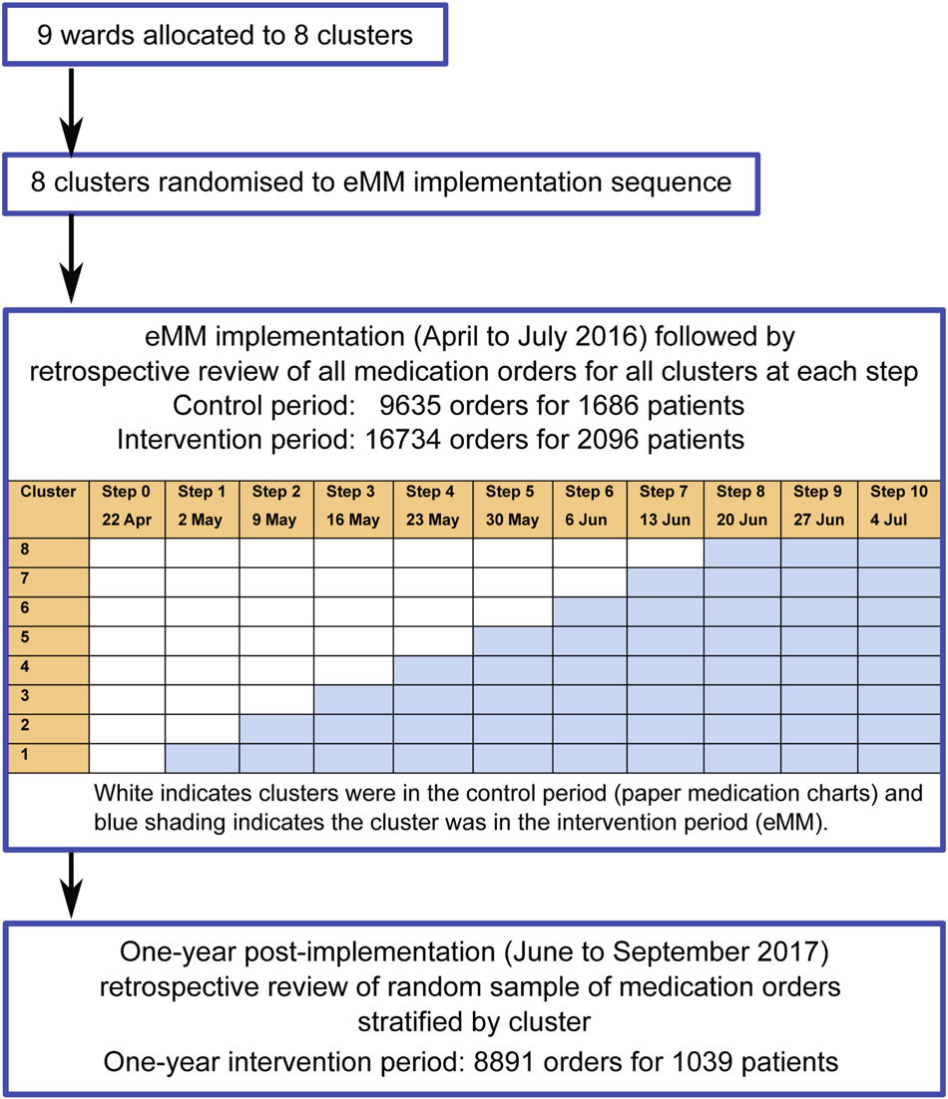
Flow diagram of stepped-wedge cluster randomised controlled trial and one-year follow-up with retrospective collection of prescribing errors.

A SWCRCT design was selected as it was not feasible to undertake individual patient randomisation. As the hospital had decided to implement the eMM across the hospital, there was no option of conducting a cluster randomised controlled trial (CRCT) where only some wards would be randomised to receive the intervention with others randomised to remain using paper medication charts. Given that wards treat different types of patients, such a CRCT would also introduce a bias when comparing intervention and control wards. A SWCRCT design allows clusters to be their own controls and thus data generated from both control and intervention periods can be compared, and hence reduce bias^33^. The SWCRCT, conducted over a period of 11 weeks (Fig. 1), allowed comparison of prescribing errors occurring in the control period (when paper medication charts were used) with those occurring when the eMM was implemented (intervention period). The SWCRCT allowed assessment of the short-term effects of the intervention on prescribing error rates during this first 70 days of eMM use at the hospital.

All inpatients during the study periods were included, except those in oncology and intensive care units, as the hospital determined that eMM implementation was not possible on these wards as part of the SWCRCT.

Nine wards were grouped into eight clusters prior to randomisation. Two wards were randomised together as there was direct physical access from one ward to the other, and they treated similar patients. Researchers, blinded to cluster/ward identity and independent of the hospital, randomly assigned (using a computer number generator) the sequence of cluster eMM implementation. The trial had 11 steps of one-week duration (Fig. 1). The hospital determined the one-week step between cluster implementations. Patients could be in different steps and/or clusters due to ward transfers or readmissions.

Following the publication of the trial protocol^32^, additional data were collected one-year post-eMM implementation to allow comparison of prescribing error rates between the control period and one-year post-eMM.

Medication administration error data were collected using direct observation during the SWCRCT and will be reported in a separate publication, as will the impact of eMM on patient length of stay. The second planned hospital site was not able to participate in a SWCRCT as originally planned in the protocol due to changes to the implementation plan by the hospital.

The trial was registered with the Australian New Zealand Clinical Trials Registry (ANZCTR) 370325. Registered 17 October 2016, and received ethical approval from the Sydney Children’s Hospital Network Human Research Ethics Committee (HREC/15/SCHN/370). Individual patient consent to access retrospective medication and clinical records was waived.

### The control and intervention

In the control period, medication orders were hand-written on paper charts. During the SWCRCT, clusters sequentially implemented the eMM. The intervention was an eMM module added to the hospital’s existing clinical information system (Cerner Corporation). The eMM allows electronic prescribing, recording of drug dispensing, drug administration and medication reconciliation and monitoring. The eMM contained passive and active decision support, e.g. guidelines, policies, protocols, order-sets, order sentences, dose-range limits, therapeutic drug monitoring and related pathology parameters, drug–drug interaction alerts, and dosage calculators. All wards had access to the same decision support. Specialised pain management prescribing remained on paper, as did sliding scale insulin and insulin pump orders.

The hospital organised an extensive staff training programme, in how to use the eMM system, which prescribers were required to complete. Training was provided for staff on each ward two weeks prior to implementation, adopting a “just in time” training approach. Multiple online resources were made available to assist with specific processes^34^. Training was classroom-based with further self-directed modules. Junior medical officers, who complete the majority of prescribing tasks, were required to demonstrate competency during the class via workbook exercises and only then allowed access to the system. Further, during the implementation, support staff were available on individual wards to assist with implementation and trouble-shooting. On the first day of implementation on each ward, paper medication charts were copied to the eMM system. All new patients entering the ward from that time onwards were entered into the eMM system. If patients were transferred to a ward without the eMM system, their medications would be transferred to a paper medication chart. Once the ward implemented the eMM, it was mandatory for staff to prescribe medications using the system. If an order was not placed in the system, the pharmacy could not dispense and a nurse could not administer the medication. All paper prescription pads and paper medication charts were removed from the wards. Downtime medication charts were available in the event of a downtime, but these were stored separately.

### Prescribing error data collection

Medication orders for inpatients in all study periods (April–July 2016; June–September 2017) were reviewed retrospectively by one of three clinical pharmacists, independent from the hospital, to identify and classify clinical and procedural prescribing errors^35^ (Supplementary Table 5). The order in which records were reviewed was randomised across all wards. All errors identified by the pharmacists were rated for their potential harm severity (to identify potential adverse drug events [ADEs]) using a five-point scale based on an adapted version of the NCC MERP Index for categorizing medication errors^36^ (Supplementary Table 6). Any documentation which indicated that staff had detected an error (e.g. note of an order correction; a case conference with parents) was recorded. Details of all medications ordered (date of prescribing, medication name and route) for each patient admission were recorded to provide denominators for error rate calculations.

Interim analysis of procedural errors was conducted after medication charts of 2000 admissions had been audited. Collection of procedural errors ceased because of the high frequency and time taken to record these errors. In total, procedural prescribing errors were collected for a random sample of 2591 patients (18,139 medication orders), which accounted for 68.5% of patients across the entire 11-week SWCRCT. Procedural errors were not collected during the one-year follow-up.

A random stratified sample of patient records in the eight clusters was obtained one year from the date of last cluster implementation (between 20 June 2017 and 20 September 2017). Stratification was conducted by cluster/ward. Number of patients for each ward was decided based on the distribution of patients admitted in study wards. Hence the number per ward was different. Patients were then randomly selected using a random number generation code (SAS).

### Training and inter-rater reliability assessment

The prescribing error definitions and detailed guidelines for how to review records and classify errors were developed based upon an existing error classification applied in studies in adult hospitals by the research team^35^. This classification was modified to account for differences in paediatric prescribing informed by hospital prescribing guidelines (Supplementary Table 5).

Training in the use of the error classification involved pharmacist reviewers applying the classification to the same subsets of patient records and then meeting to discuss any differences in outcomes. Once training was complete, inter-rater reliability between the three pharmacist reviewers (the most experienced, selected as the gold standard) was conducted to ensure all reviewers had good agreement in classifying prescribing errors and potential severity of those errors. The pharmacist reviewers obtained a minimum Cohen’s kappa score of 0.77 on error type and 0.76 on severity level before they commenced independent data collection. The second round of inter-rater reliability was conducted a year later (minimum kappa 0.95 and 1.0, respectively).

### Assessing actual harm associated with clinical prescribing errors

A multi-disciplinary harm assessment panel (comprising a paediatrician, paediatric nurse and pharmacist and/or clinical pharmacologist) met on 12 occasions to determine actual harm associated with errors (i.e. actual ADEs). The medical records of patients with errors with a potential harm severity score of ≥3 were reviewed by clinically trained members of the research team, prior to cases being referred to the harm panel, to exclude errors not administered to patients (e.g. order with dose error that was not administered; or duplicate orders not administered).

Detailed case reports (outlining a patient’s medical history, prescribing errors, other medications, signs and symptoms, changes to treatment/care before and after the error, and relevant test results) were prepared by clinically trained members of the research team. The harm panel members each reviewed the cases individually, and then met to gain consensus about any actual harms and the severity. Some patients experienced multiple errors (Supplementary Table 3). Where harm was identified, the severity was classified using the Harm Associated with Medication Error Classification (HAMEC)^37^ and the probability that the error caused the harm was classified using a modified version of the WHO Uppsala Monitoring Centre categories^38^ (Supplementary Tables 7 and 8). Overall, this was a laborious process which commenced in 2019, and completed in 2021. Reduced access to clinicians for the panels due to the pandemic slowed progress.

### Sample size calculations

As outlined in our protocol, the expected reduction in overall prescribing error rate was 60%, from 4.06 errors per admission (SD = 5.27) to 1.62 (SD = 2.87) with an estimated intraclass correlation coefficient (ICC) of 0.06. Collecting data on eight clusters would allow detection of a minimum change of 20% for overall errors with 100% power and 42% change for potential ADEs with 83% power (for two-sided tests; α < 5%). At each step, records for 112 patient admissions were estimated to be required, totalling 1232 across the study.

The one-year follow-up sample size was calculated based on data from 1817 patients generated during the SWCRCT. Using these data, we calculated a clinical prescribing error rate of 0.24/patient-day (SD = 0.78) with an ICC of 0.04 for eight clusters. To detect a 35% change one-year post-eMM, we required 1230 patient records for the one-year post-eMM follow-up sample (for a two-sided test; 80% power; α < 5%).

### Analysis

The study involved comparison of error rates for three periods: (1) Control, when prescribing occurred on paper medication charts during the SWCRCT; (2) Intervention, first 70 days of eMM use measured during the SWCRCT; and (3) one-year post-eMM, when prescribing using the eMM had been in place for one year on all wards. Outcome measures are listed in Box 1.

We applied an intention to treat analysis, with all orders analysed according to whether the ward was using eMM or paper medication charts. Crude error rates per 100 orders with 95% confidence intervals (CIs) were estimated from simple Poisson models. Errors per 100 orders in the SWCRCT, i.e. control and intervention periods, were modelled using generalised linear mixed-effects negative binomial models accounting for the stepped-wedge design^39,40^. The dependent variable was the number of errors per patient-day, with an offset of the logarithm of the number of orders per patient-day. Random effects terms were included for cluster (unit of randomisation) and patient (to account for admissions over multiple days). As observations for each day were available, we modelled the secular trend as continuous using restricted cubic splines with 4 degrees of freedom^40,41^. As the average patient age was younger in the paper period (because of the order of eMM rollout to wards with different patient profiles), we adjusted for age in months as a restricted cubic spline with 2 degrees of freedom (Supplementary Table 9). We used splines for the secular trend and patient age to avoid assuming linear associations with errors. Knots were placed at recommended quantiles of the variables^41^. An indicator variable was included for eMM vs paper. This model assumes a constant effect of the intervention throughout the study period and across all clusters. From the models, we estimated incidence rate ratios (IRRs) for eMM use (intervention) vs paper (control). We used similar models without the secular trend to estimate IRRs for one-year post-eMM vs paper. Clinical and procedural errors were modelled separately, with additional models for errors with potential severity score ≥3 (i.e. potential ADE) and for orders involving high-risk drugs. To model rates of individual error types, we used the same model specifications used for the main results, but with Poisson instead of negative binomial distribution as there was no overdispersion at this level and negative binomial models failed to converge.

We used a *χ*^2^ test to assess any difference in the proportion of errors detected by staff in the paper and eMM periods. Data management was performed in SAS version 9.4, and analysis in R version 4.2. Model estimation used glmmTMB^42^, and model fit was assessed using DHARMa^43^.

Box 1: Outcome measures Primary outcome measures

- Clinical prescribing error rates
- Potential adverse drug event rates (potential Adverse Drug Event [ADE], potential harm severity score ≥3)

Secondary outcome measures

- Clinical prescribing error rates by type, and those associated with high-risk^a^ medications
- Error detection rates - Prescribing errors where there was documentation in patient records indicating that errors had been detected by staff
- Actual ADEs (Prescribing errors that resulted in harm, injury or required responsive action (only for the SWCRCT)
- Procedural prescribing error rates (only for the SWCRCT)

^a^High-risk medications were defined by the hospital as anti-infectives, potassium and other electrolytes, insulin, narcotics/opioids and sedatives, chemotherapy agents and heparin and other anticoagulants. (Sydney Children’s Hospital Network. High risk medicine register, 2015.)

### Reporting summary

Further information on research design is available in the Nature Research Reporting Summary linked to this article.

## Supplementary information

Supplementary Material

Reporting Summary

## Data Availability

This study used individual patient health data that cannot be shared without ethical approval. This also precludes the sharing of aggregated datasets. Analysis datasets are stored according to the ethical approval and access can only be provided to researchers who have received approval from the ethics committee.

## Code availability

The code used for preparing and analysing the data is available from the corresponding author on reasonable request.
