# Supplementary material for: Short- and long-term effects of an electronic medication management system on paediatric prescribing errors
Source: NPJ Digit Med. 2022 Dec 13;5:179. doi: 10.1038/s41746-022-00739-x (PMC9747795; doi:10.1038/s41746-022-00739-x)
Supplement: Supplementary file 1 — Supplementary Material [file 41746_2022_739_MOESM1_ESM.pdf]

Supplementary Tables

Short- and long-term effects of an electronic medication management system on paediatric prescribing errors

Westbrook JI et al

Supplementary Table 1 Changes in procedural prescribing error rates

	Control - Paper		Intervention – First 70 days of eMM use			P value [3]
	N	Errors/ 100 orders (95% CI) [2]	N	Errors/ 100 orders (95% CI) [2]	IRR: eMM vs Paper (95% CI) [3]	
Procedural errors[1]	6698 orders (1162 patients)		11441 orders (1429 patients)			
All procedural errors	7652	114.24 (111.71-116.83)	7699	67.29 (65.81-68.81)	0.65 (0.61-0.70)	<0.001
Procedural errors, potential severity 3+	127	1.90 (1.59-2.26)	180	1.57 (1.36-1.82)	0.78 (0.46-1.32)	0.35
Patients with high-risk drug orders	834 orders (526 patients)		1534 orders (798 patients)			
Procedural errors in high-risk drug orders	942	112.95 (105.96-120.40)	1101	71.77 (67.66-76.14)	0.62 (0.53-0.72)	<0.001

[1]Procedural errors were assessed from a random sample comprising 68.5% of patients during the entire SWCRCT period. [2]Crude error rates and 95% CIs are from simple Poisson models. [3]Incidence Rate Ratio (IRR), 95% CIs and P values from generalised linear mixed models accounting for patient age, study time (immediate post-eMM only), and clustering by ward (unit of randomisation) and patient.

Supplementary Table 2 Procedural prescribing error rates by error type

Error type	Control - Paper		Intervention – First 70 days of eMM Use		IRR, eMM vs Paper
	Errors	Errors/100 orders (95% CI)	Errors	Errors/100 orders (95% CI)	
Procedural prescribing errors	*1162 patients (6698 orders)*		*1429 patients (11441 orders)*		
Unclear order	*747*	*11.2 (10.4-12.0)*	*598*	*5.2 (4.8-5.7)*	*0.6 (0.5-0.7)*
Unclear or ambiguous	314	4.7 (4.2-5.2)	311	2.7 (2.4-3.0)	0.8 (0.6-1.1)
Illegible	396	5.9 (5.4-6.5)	127	1.1 (0.9-1.3)	0.2 (0.2-0.3)
Contradictory information	37	0.6 (0.4-0.8)	160	1.4 (1.2-1.6)	2.9 (1.4-5.8)
Incomplete order	*3059*	*45.7 (44.1-47.3)*	*6256*	*54.7 (53.3-56.1)*	*1.3 (1.2-1.4)*
Missing duration (antimicrobial or paracetamol)	1458	21.8 (20.7-22.9)	2239	19.6 (18.8-20.4)	1.0 (0.9-1.1)
No max dose for PRN	1264	18.9 (17.9-19.9)	3726	32.6 (31.5-33.6)	1.7 (1.6-2.0)
Other	337	5.0 (4.5-5.6)	291	2.5 (2.3-2.9)	0.6 (0.5-0.8)
Illegal order	*115*	*1.7 (1.4-2.1)*	*63*	*0.6 (0.4-0.7)*	*0.3 (0.1-0.8)*
Missing signature	36	0.5 (0.4-0.7)	8	0.1 (0.0-0.1)	0.1 (0.0-1.2)
Legal requirements not met	79	1.2 (0.9-1.5)	55	0.5 (0.4-0.6)	0.2 (0.0-1.5)
Not best practice	*3701*	*55.3 (53.5-57.1)*	*750*	*6.6 (6.1-7.0)*	*0.2 (0.2-0.2)*
Unapproved abbreviation	2555	38.1 (36.7-39.7)	500	4.4 (4.0-4.8)	0.2 (0.2-0.2)
Altered order	595	8.9 (8.2-9.6)	127	1.1 (0.9-1.3)	0.2 (0.1-0.2)
Brand name used	395	5.9 (5.3-6.5)	105	0.9 (0.8-1.1)	0.3 (0.2-0.4)
Multiple routes used	59	0.9 (0.7-1.1)	7	0.1 (0.0-0.1)	0.1 (0.0-0.5)
Other	97	1.4 (1.2-1.8)	11	0.1 (0.1-0.2)	0.1 (0.0-0.5)
NIMC post-eMM	*30*	*0.4 (0.3-0.6)*	*32*	*0.3 (0.2-0.4)*	*0.2 (0.0-1.5)*

NIMC – National Inpatient Medication Chart

IRR Incidence Rate Ratio

Supplementary Table 3 Clinical prescribing error types where the number of errors precluded modelling

Error type	Control - Paper (n=9635 orders)		Intervention – First 70 days of eMM (n=16734 orders)			Intervention - One-year post-eMM (n=8891 orders)		
	No. Errors	Errors/100 orders (95% CI)[1]	No. Errors	Errors/100 orders (95% CI)[1]	IRR[2] eMM vs Paper	No. Errors	Errors/100 orders (95% CI)[1]	IRR[2] one-year post-eMM vs Paper
Wrong formulation	7	0.07 (0.03-0.15)	36	0.22 (0.16-0.30)	0.74 (0.15-3.74)	7	0.08 (0.04-0.17)	---
Wrong timing	4	0.04 (0.02-0.11)	34	0.20 (0.15-0.28)	---	1	0.01 (<0.01-0.08)	---
Wrong strength	3	0.03 (0.01-0.10)	10	0.06 (0.03-0.11)	---	9	0.10 (0.05-0.19)	---
Wrong patient	2	0.02 (0-0.07)	2	0.01 (<0.01-0.04)	---	0	0 (0-0.04)	
Wrong rate	2	0.02 (0.01-0.08)	2	0.01 (<0.01-0.05)	---	3	0.03 (0.01-0.10)	---
No longer indicated	0	0 (0-0.04)	3	0.02 (<0.01-0.05)	---	0	0 (0-0.04)	---

[1]Crude error rates and 95% CIs are from simple Poisson models. [2]Adjusted Incidence Rate Ratio (IRR) and 95% CIs are from generalised linear mixed models adjusting for study time (immediate post-eMM model only), patient age, and clustering by ward and patient. --- indicates too few errors for modelling.

Supplementary Table 4: Clinical prescribing errors associated with actual harms

**Harm no.**	**No. of errors involved**	**Study cohort**	**Error type/s involved**	**Harm rating**	**Causality rating**
1	1	Control - Paper	Dose error	Moderate	Probable
2	2	First 70 days of eMM	Wrong drug; wrong frequency	Minor	Certain
3	1	Control - Paper	Dose error	Moderate	Certain
4	1	First 70 days of eMM	Dose error	Moderate	Probable
5	1	First 70 days of eMM	Dose error	Minor	Certain
5	1	Control - Paper	Dose error	Minor	Possible
7	1	First 70 days of eMM	Dose error	Moderate	Probable
8	9	First 70 days of eMM	Dose error; wrong route	Minor	Probable
9	1	Control - Paper	Dose error	Minor	Certain
10	3	Control - Paper	Wrong route	Moderate	Possible
11	1	First 70 days of eMM	Dose error	Minor	Possible
12	6	First 70 days of eMM	Wrong route	Moderate	Possible
13	1	First 70 days of eMM	Dose error	Moderate	Possible
14	2	Control - Paper	Duplicated drug therapy	Minor	Probable
15	1	Control - Paper	Dose error	Serious	Probable
16	3	First 70 days of eMM	Wrong route	Moderate	Possible
17	1	Control - Paper	Drug-drug interaction	Moderate	Possible
18	1	First 70 days of eMM	Dose error	Minor	Possible
19	1	Control - Paper	Dose error	Moderate	Probable
20	1	Control - Paper	Dose error	Moderate	Possible
21	1	Control - Paper	Dose error	Moderate	Probable

The panel determined that five cases presented to the panel could not be assessed for harm due to insufficient documentation in patients' medical records.

Supplementary Table 5: Prescribing error classification and definitions

Clinical prescribing errors			
Error category	Sub-category	Definition	Examples
Wrong patient		A medication is prescribed for the wrong patient.	
Wrong drug	Drug-disease interaction	The drug or IV fluid is contraindicated for a co-existing condition.	• NSAID prescribed to an asthmatic patient.<br>• Gentamicin is prescribed to a patient with a history of auditory toxicity caused by an aminoglycoside.
	Incompatible diluent	An IV drug is prescribed with an incompatible diluent (if prescribed).	• Amphotericin B is prescribed with sodium chloride 0.9% as diluent.
	Other	A wrong drug error that cannot be categorized as drug-disease interaction and incompatible diluent.<br>**Exclusion:**<br>Generic or formulary substitution.	• Hyoscine hydrobromide is prescribed for CF patient when hyoscine butylbromide is indicated.<br>• Fluticasone/salmeterol inhaler is prescribed for a patient without a respiratory condition, such as asthma.<br>• Look-alike, sound-alike errors.
Wrong formulation		The wrong dosage form of a medication is prescribed.	• An extended-release tablet is prescribed when an immediate release form is required.<br>• Oral formulation prescribed for IV administration.
Wrong strength		1. The prescribed drug strength is incorrect.<br>2. The concentration of an IV infusion is prescribed incorrectly.*	• Amoxicillin/clavulanic acid 1:4 ratio prescribed with dose and frequency for 1:7 ratio<br>• Wrong volume of diluent for IV drug i.e. concentration too low or too high

		3. The prescribed dose does not exist or would not be able to be obtained from the current dose form.<br><br>**Exclusion:**<br><br>Wrong strength which can directly result in either overdose or underdose should be categorised under "Wrong Dose".<br><br>*Incorrect concentration of infusions was classified as any concentration above or below the limits of reference guidelines	• Dose unit such as suppository prescribed with strength that does not exist
Wrong dose	Overdose	1. The prescribed medication dose or IV fluid volume is >10% higher than that recommended for the condition, taking into account the patient's age and weight.<br><br>2. The dosing fails to take into account renal and liver function.<br><br>3. A dose is not altered in response to abnormal drug serum levels or laboratory tests.<br><br>**Exclusion:** Doses where local protocols allow for rounding to the nearest measurable dose e.g. quarter tablet for spironolactone	• Maintenance oral paracetamol dose prescribed at 30mg/kg every six hours for a 2-month-old infant.<br>• Clonidine prescribed at 200microg bd for a 10-year-old with ADHD (reference states a max dose of 300mcg/day in 2-3 doses).
	Under dose	1. The prescribed medication dose or IV fluid volume is >10% lower than that recommended for the condition, taking into account the patient's age and weight.<br><br>2. The dosing fails to take into account renal and liver function.<br><br>3. A dose is not altered in response to abnormal drug serum levels or laboratory tests.<br><br>**Exclusion:** Doses where local protocols allow for rounding to the nearest measurable dose e.g. quarter tablet for spironolactone	• Initial dose of gentamicin for a 6-month-old infant with normal renal function is below recommended dose of 7.5mg/kg once daily.<br>• hydrocortisone IV bolus 25mg tds prescribed for a 18 year old patient for acute adrenal insufficiency (reference advises dose of 50–100 mg every 6–8 hours)

	Wrong unit	The prescribed medication dose contains the wrong unit which results in either over or under dose.<br><br>The unit of measure used does not correspond with the dosage form or recommendations for safe prescribing.	• Thyroxine 100 “mg” mane is charted instead of “mcg” which would result in overdose.<br>• Pancreatic enzymes prescribed as capsules or scoops instead of ‘units’
	Dose unverified	The prescribed medication dose cannot be verified from reference sources.	• Clinical trial drugs without standard dosage guidelines.<br>• Doses where references do not contain dosing recommendation for condition being treated.<br>• Drug prescribed in mL where multiple strengths of liquid formulation are available and dose cannot be determined from order
Wrong route		A medication is prescribed via an incorrect route of administration.	• IV medication is prescribed orally except for off-label use.<br>• Left eye was written instead of right eye.<br>• PO route is charted for nil by mouth patient on PEG.<br>• IV bolus prescribed for medication that requires intermittent infusion.
Wrong frequency		The prescribed frequency of administration of a drug is outside the recommended range.<br><br>The frequency is acceptable but the total number of doses exceeds the maximum daily recommended dose	• Ceftriaxone TDS<br>• Paracetamol 15mg/kg 4- hourly prn missing maximum daily dose information (60mg/kg/day)<br>• Daily dose divided into multiple doses for drug usually given once daily<br>• PRN order with correct frequency and max dose combined with regular order to push over limit e.g. regular ondansetron mane and TDS prn (usually maximum 3 times/day)

Wrong rate	Too fast	An IV rate or the basal rate of infusion falls above the recommended range.<br>**Exclusion:**<br>IV medications requiring TDM for dose adjustments e.g. heparin and vancomycin continuous infusion, review is to be guided by TDM level rather than dose and rate.	
	Too slow	An IV rate or the basal rate of infusion falls below the recommended range.<br>**Exclusion:**<br>IV medications requiring TDM for dose adjustments e.g. heparin and vancomycin continuous infusion, review is to be guided by TDM level rather than dose and rate.	
Wrong timing		A medication is prescribed at the wrong time of day.	• Methylphenidate prescribed in the evening instead of morning.<br>• NSAID prescribed to take before food.<br>• Pre-medication prescribed to prevent infusion reaction scheduled after infusion
No longer indicated		1. A drug is continued following a clinically significant adverse drug reaction.<br>2. A drug that is no longer indicated is re-ordered.<br>3. A drug which should have been discontinued has not been ceased.	• Clonazepam continues to be prescribed following episode of respiratory depression.<br>• Ranitidine liquid is re-ordered following recovery from symptomatic GORD.<br>• Continuation of antibiotic use following recovery from an infection.
Duplicated drug therapy		1. Same drug prescribed twice as two active orders at the same time.<br>2. The same drug is prescribed twice, as a single agent and as a combination product.	• Paracetamol prescribed for both prn and regular doses.<br>• Humalog and Humalog Mix25 which both contain ultra-shorting-acting insulin are charted

		3. Two drugs are prescribed for the same indication when only one is necessary (including two drugs from the same class with the same clinical effect).	• Nurse/patient-controlled analgesia prescribed at same time as oral opiates without instruction on when to administer each order
Drug-drug interaction		Two or more prescribed drugs have a clinically significant interaction	• Gentamicin and frusemide are prescribed together, increasing the risk of nephrotoxicity and ototoxicity.<br>• Ondansetron, droperidol and ciprofloxacin are prescribed together which are known to cause QT interval prolongation.
Allergy and adverse drug reaction		A drug is prescribed for a patient with a documented clinically significant adverse reaction to that drug or that class of drugs. Includes drug classes with know cross-reactivity e.g. penicillins and cephalosporins	• Amoxicillin prescribed for a patient who is allergic to penicillin.<br>• Morphine is prescribed for a patient with documented history of itch from morphine.
Inadequate monitoring		The prescriber fails to order appropriate and timely clinical or laboratory tests to assess the patient's response to prescribed therapy.<br>*If adequate laboratory tests are ordered, but the results are not acted upon accordingly, this may be classed as wrong dose or frequency.	• Insulin is prescribed without requesting blood glucose monitoring.<br>• IV potassium is prescribed at infusion rate >0.4mmol/kg/hour without ECG monitoring and frequent K levels check.<br>• Vancomycin is ordered without TDM and renal function monitoring.
**Procedural errors**			
**Error category**	**Sub-category**	**Definition**	**Examples**
Unclear order	The prescription is unclear or ambiguous	The prescription is unclear or ambiguous.	• Clotrimoxazole topical BD was prescribed (the prescriber was confused between cotrimoxazole and clotrimazole).

			• Warfarin variable dose prescribed as prn dose.<br>• Oxycodone 5mg prn with a frequency of “as indicated” without a specified frequency.<br>• Paracetamol is charted on eMM with a free text indication for ‘nausea and vomiting’.<br>• Liquid medication written in mL when it should be prescribed in mg (excludes vitamins liquids etc. where volume dosing is more appropriate).
	Illegible handwriting	Any element of the hand-written prescription is illegible.	
	Order contains contradicting information	The order contains additional comments that contradict the medication order.	• An IV order with an instruction to swallow whole.<br>• Captopril 2.5mg daily with instructions ‘Dissolve 1x5mg tablet in 5mL of water and give 2mL)’.
Incomplete order	No defined treatment duration for antibiotics or paracetamol	An antibiotic is prescribed without a specified course length.<br>A paracetamol order is prescribed without a maximum duration of therapy or review timeframe as per hospital guidelines.<br><br>**Exclusion:**<br>Long-term antibiotic prophylactic use eg. Bactrim and azoles used in immuno-compromised patient with haematology disorders.	• No specified treatment duration of penicillin for tonsillitis.
	No max dose for PRN med	A maximum dosage is not prescribed for a PRN medication order.	• Oxycodone prn prescribed without stating the max dose to be administered per 24 hours.

	Other	Other incomplete orders apart from “no stop date for antibiotics/paracetamol” and “No max dose for PRN med”.<br><br>The order does not include all the necessary drug information including prescribing date, drug name, strength (if applicable), formulation (if applicable), dose, route, frequency, the diluent for the injectable (IV, IM), duration of time and/or rate of infusion (IV infusion).<br><br>**Exclusion:** Indication or dose calculation information only fields	• Nasal decongestant prescribed without maximum duration of use.<br>• Dose written as mg/kg without calculating total dose in mg for patient weight.
Legal error	Missing signature	The order is not signed. Phone/verbal orders not signed by the ordering prescriber within 24 hours.	• Missing prescriber’s signature within 24 hours of phone order when order has been administered.
	Use of NIMC or eMM in wrong place	When the paper medication chart is used after eMM implementation.<br><br>When the eMM is used to prescribe medication for which prescribing remains on paper.<br><br>**Exclusion:** The use of paper charts in non-eMM areas of the hospital (e.g. imaging) or during system downtime.	• Ongoing use of NIMC (paper chart) during post-eMM phase.<br>• Prescribing of nurse-controlled analgesia protocol in the eMM instead of the paper pain chart.
	Unapproved abbreviation	The use of abbreviations not approved by local and government policy.	• 'q4h' to convey 'every four hours' is an unapproved abbreviation according to hospital medication handling policy and Australian Commission on Safety and Quality in Health Care
	Altered order	The strength, dose, route or frequency of an existing handwritten medication order has been altered (such a change legally requires the entire order to be recharted).	• Changing the dose of an order without ceasing the order and re-prescribing with a new dose
	Brand name used	Use of brand names instead of generic names.	• Seretide Accuhaler (fluticasone/salmeterol 250/50)

		**Exclusion:** combination drugs e.g. ticarcillin/clavulanic acid; non-bioequivalent drugs eg. cyclosporin, sodium valproate and warfarin; complementary medicines eg. multivitamins and supplements; formulations where the use of generic names may be cause confusion).	vs Seretide MDI (fluticasone 250/25)
	Multiple routes used	**Exclusion:** route that may be used interchangeably e.g. PO/NG and drugs where local policy allows nurses to determine route based on patient parameters e.g. ondansetron IV/PO for nausea and vomiting.	• Prescribing levetiracetam with route IV/oral
	Legal requirements not met	An aspect of the prescription does not comply with the law, the NSW Department of Health or the hospital policy (and has not been assigned as an unclear order or incomplete order).	• Incorrect order date charted in the wrong month or day.<br>• Dose for opiates required to be written in words and numbers for hand-written orders e.g. oxycodone 2mg (two milligrams)
	Others	Any other legal or policy driven prescribing requirement not outlined above.	• Slow-release box not ticked for slow-release formulation e.g. MS Contin (Legal error-other)

Supplementary Table 6 Prescribing error potential harm severity rating scale[1]

1. MINIMUM: An error occurred with no or minimal potential to cause harm to the patient and no need for a change in monitoring or intervention
2. MINOR: An error occurred which has the potential to cause temporary harm to the patient and would require monitoring
3. MODERATE: An error occurred which has the potential to cause temporary harm to the patient and would require intervention
4. MAJOR: An error occurred which has the potential to cause permanent harm to the patient and would require intervention
5. SERIOUS: An error occurred which has the potential to result in patient death and/or would require life support interventions

Monitoring: Any change in care assessment pattern from the usual or standard level of care including age appropriate clinical assessment of vital signs, neurological status, fluid balance, pain score, oxygen saturation and cardiac monitoring.

Intervention: Any intervention including diagnostic pathology, medical imaging, cardiac investigations, medications, or general medical/surgical treatment and nursing care.

The NCC-MERP Index for Categorizing Medication Errors scale was designed to assess actual patient harm that occurred, and not potential harm. We adapted the NCC-MERP[1] scale to assess ‘potential harm’ by collapsing some of the categories, resulting in a five-point scale. For example, Category H in the NCC-MERP scale is ‘an error occurred that required interventions to sustain life’ and category I is ‘an error occurred that may have contributed to or resulted in the patient’s death’. Making this kind of distinction is not possible when assessing ‘potential’ harm from an error, as it is very dependent on the actions a hospital takes or does not take, and whether an error is detected in a timely manner. Thus, our most severe error category had the description ‘An error occurred which has the potential to result in patient death and/or would require life support interventions’.

[1] Adapted from National Coordinating Council for Medication Error Reporting and Prevention. NCC MERP Index for categorizing medication errors, <www.nccmerp.org> (2001).

Supplementary Table 7 Assessment of actual harm - Harm Associated with Medication Error Classification[1] (HAMEC)

Actual harm rating	Description
Minor	There was potential for minor, non-life threatening, temporary harm that may or may not require efforts to assess for a change in a patient's condition such as monitoring[a]. These efforts may or may not have potentially caused minimal increase in length of care (< 1 day)
Moderate	There was potential for minor, non-life threatening, temporary harm that would require efforts to assess for a change in a patient's condition such as monitoring[2], and additional low-level change in a patient's level of care[b] such as a blood test. Any potential increase in the length of care is likely to be minimal (< 1 day)
Serious	There was potential for major, non-life threatening, temporary harm, or minor permanent harm[4] that would require a high level of care[3] such as the administration of an antidote. An increase in the length of care of ≥ 1 day is expected
Severe	There was potential for life-threatening or mortal harm, or major permanent harm[4] that would require a high level of care[3] such as the administration of an antidote or transfer to intensive care. A substantial increase in the length of care of > 1 day is expected

[1] Gates PJ, Baysari MT, Mumford V, et al. Standardising the Classification of Harm Associated with Medication Errors: The Harm Associated with Medication Error Classification (HAMEC). *Drug Saf* 2019;42(8):931-39.

[2] Monitoring refers to the minimally intrusive observation of the patient's condition over time. Observations are typically made for urine output, general level of consciousness, or vital signs including heart or breathing rate

[3] Level of care refers to the degree of active treatments that are initiated in response to actual or potential change in the patient's condition

[4] Permanent harm is such that, as a consequence of the drug event, the patient would require ongoing care, or experience an ongoing disability, beyond the index admission

Supplementary Table 8 Causality assessment categories used to classify the likelihood the harm was caused by the error[1]

Rating	Conditions for rating
Certain	Event or laboratory test abnormality with plausible time relationship to intake of a drug<br>Cannot be explained by disease or other drugs<br>Event definitive pharmacologically or phenomenologically
Probable	Event or laboratory test abnormality, with reasonable time relationship to drug intake<br>Unlikely to be attributed to disease or other drugs
Possible	Event or laboratory test abnormality, with reasonable time relationship to drug intake<br>Could also be explained by disease or other drugs
Unlikely	Event or laboratory test abnormality, with a time to drug intake that makes a relationship improbable<br>Disease or other drugs provide plausible explanations

[1]Adapted from the WHO Uppsala Monitoring Centre categories. Uppsala Monitoring Centre. The use of the WHO-UMC system for standardised case causality assessment Sweden: Uppsala Monitoring Centre, 2018.

Supplementary Table 9 Generalised linear mixed model outputs

**Model outputs for clinical prescribing errors**									
	**Crude model**			**Stepped wedge cluster RCT model**			**Pre-post model 1-year post eMM**		
Term	Estimate	SE	P	Estimate	SE	P	Estimate	SE	P
Intercept	-1.93	0.03	<0.001	-2.31	0.10	<0.001	-2.31	0.09	<0.001
eMM (ref = Paper)	0.19	0.03	<0.001	0.05	0.07	0.454	0.00	0.05	0.965
1-year post-eMM	-0.21	0.04	<0.001				-0.45	0.06	<0.001
Study day spline term 1				0.04	0.11	0.706			
Study day spline term 2				-0.08	0.11	0.469			
Study day spline term 3				-0.30	0.18	0.095			
Study day spline term 4				-0.08	0.09	0.400			
Patient age spline term 1				0.48	0.13	<0.001	0.44	0.12	<0.001
Patient age spline term 2				0.31	0.06	<0.001	0.31	0.06	<0.001
Random effects				SD					
Cluster				0.16			0.17		
Patient				0.70			0.67		

**Model outputs for clinical errors with ≥3 severity**									
	**Crude model**			**Stepped wedge cluster RCT model**			**Pre-post model 1-year post eMM**		
Term	Estimate	SE	P	Estimate	SE	P	Estimate	SE	P
Intercept	-3.31	0.05	<0.001	-4.87	0.20	<0.001	-4.98	0.16	<0.001
eMM (ref = Paper)	0.28	0.06	<0.001	0.48	0.12	<0.001	0.22	0.09	0.018
1 year post-eMM	-0.09	0.08	0.28				-0.08	0.13	0.554
Study day spline term 1				0.04	0.22	0.872			
Study day spline term 2				-0.65	0.22	0.003			
Study day spline term 3				-0.69	0.35	0.047			
Study day spline term 4				-0.36	0.18	0.049			
Patient age spline term 1				0.07	0.30	0.805	0.17	0.27	0.529
Patient age spline term 2				0.36	0.15	0.016	0.47	0.13	0.000
Random effects				SD					
Cluster				0.00			0.00		
Patient				1.81			1.78		

Model outputs for clinical errors for high risk[1] drugs									
	**Crude model**			**Stepped wedge cluster RCT model**			**Pre-post model 1-year post eMM**		
Term	Estimate	SE	P	Estimate	SE	P	Estimate	SE	P
Intercept	-1.64	0.07	<0.001	-2.52	0.19	<0.001	-2.46	0.14	<0.001
eMM (ref = Paper)	-0.12	0.08	0.15	-0.13	0.14	0.354	-0.19	0.11	0.068
1 year post-eMM	-0.38	0.1	<0.001				-0.40	0.14	0.004
Study day spline term 1				0.09	0.26	0.737			
Study day spline term 2				-0.09	0.27	0.744			
Study day spline term 3				-0.05	0.42	0.906			
Study day spline term 4				-0.21	0.22	0.338			
Patient age spline term 1				-0.07	0.29	0.809	0.05	0.25	0.848
Patient age spline term 2				0.27	0.15	0.070	0.24	0.13	0.061
Random effects				SD					
Cluster				0.00			0.00		
Patient				1.21			1.16		

[1] High-risk medications were defined by the hospital as anti-infectives, potassium and other electrolytes, insulin, narcotics/opioids and sedatives, chemotherapy agents and heparin and other anticoagulants.

**Model outputs for procedural prescribing errors**						
	**Crude model**			**Stepped wedge cluster RCT model**		
Term	Estimate	SE	P	Estimate	SE	P
Intercept	0.13	0.01	<0.001	0.10	0.09	0.259
eMM (ref = Paper)	-0.53	0.02	<0.001	-0.43	0.04	<0.001
Study day spline term 1				-0.04	0.05	0.463
Study day spline term 2				-0.03	0.06	0.542
Study day spline term 3				-0.21	0.08	0.012
Study day spline term 4				-0.10	0.05	0.053
Patient age spline term 1				-0.10	0.06	0.108
Patient age spline term 2				-0.19	0.03	<0.001
Random effects				SD		
Cluster				0.22		
Patient				0.27		

Model outputs for procedural prescribing errors with ≥3 severity						
	**Crude model**			**Stepped wedge cluster RCT model**		
Term	Estimate	SE	P	Estimate	SE	P
Intercept	-3.97	0.09	<0.001	-7.29	0.55	<0.001
eMM (ref = Paper)	-0.19	0.12	0.11	-0.25	0.27	0.354
Study day spline term 1				0.39	0.46	0.386
Study day spline term 2				0.54	0.45	0.233
Study day spline term 3				-0.21	0.68	0.755
Study day spline term 4				-0.28	0.45	0.532
Patient age spline term 1				1.15	0.65	0.074
Patient age spline term 2				0.39	0.31	0.204
Random effects				SD		
Cluster				0.00		
Patient				3.20		

Model outputs for procedural prescribing errors for high-risk[1] medications						
	**Crude model**			**Stepped wedge cluster RCT model**		
Term	Estimate	SE	P	Estimate	SE	P
Intercept	0.12	0.03	<0.001	0.00	0.12	0.978
eMM (ref = Paper)	-0.45	0.04	<0.001	-0.48	0.08	<0.001
Study day spline term 1				0.07	0.12	0.552
Study day spline term 2				0.03	0.12	0.801
Study day spline term 3				0.00	0.18	0.982
Study day spline term 4				0.02	0.10	0.869
Patient age spline term 1				-0.15	0.11	0.182
Patient age spline term 2				-0.08	0.06	0.167
Random effects				SD		
Cluster				0.26		
Patient				0.08		

[1] High-risk medications were defined by the hospital as anti-infectives, potassium and other electrolytes, insulin, narcotics/opioids and sedatives, chemotherapy agents and heparin and other anticoagulants.
