# Supplementary material for: Short- and long-term effects of an electronic medication management system on paediatric prescribing errors
Source: NPJ Digit Med. 2022 Dec 13;5:179. doi: 10.1038/s41746-022-00739-x (PMC9747795; doi:10.1038/s41746-022-00739-x)
Supplement: Supplementary file 2 — Reporting Summary [file 41746_2022_739_MOESM2_ESM.pdf]

nature portfolio

Corresponding author(s): Johanna I Westbrook

Last updated by author(s): Nov 23, 2022

Reporting Summary

Nature Portfolio wishes to improve the reproducibility of the work that we publish. This form provides structure for consistency and transparency in reporting. For further information on Nature Portfolio policies, see our Editorial Policies and the Editorial Policy Checklist.

Statistics

For all statistical analyses, confirm that the following items are present in the figure legend, table legend, main text, or Methods section.

n/a	Confirmed	
☐	☒	The exact sample size (*n*) for each experimental group/condition, given as a discrete number and unit of measurement
☐	☒	A statement on whether measurements were taken from distinct samples or whether the same sample was measured repeatedly
☐	☒	The statistical test(s) used AND whether they are one- or two-sided<br>*Only common tests should be described solely by name; describe more complex techniques in the Methods section.*
☐	☒	A description of all covariates tested
☐	☒	A description of any assumptions or corrections, such as tests of normality and adjustment for multiple comparisons
☐	☒	A full description of the statistical parameters including central tendency (e.g. means) or other basic estimates (e.g. regression coefficient) AND variation (e.g. standard deviation) or associated estimates of uncertainty (e.g. confidence intervals)
☐	☒	For null hypothesis testing, the test statistic (e.g. F, t, r) with confidence intervals, effect sizes, degrees of freedom and P value noted<br>*Give P values as exact values whenever suitable.*
☒	☐	For Bayesian analysis, information on the choice of priors and Markov chain Monte Carlo settings
☐	☒	For hierarchical and complex designs, identification of the appropriate level for tests and full reporting of outcomes
☐	☒	Estimates of effect sizes (e.g. Cohen's *d*, Pearson's *r*), indicating how they were calculated

Our web collection on statistics for biologists contains articles on many of the points above.

Software and code

Policy information about availability of computer code

Data collection	Denominator electronic medication orders data were extracted from the hospital database (Cerner) into csv files by hospital staff. Denominator paper medication orders data were manually extracted from medical records and entered into Microsoft Excel. Error data were manually audited and extracted from the hospital systems or paper charts and entered into Microsoft Access for checking, then exported as csv files. All the xlsx and csv files (error and orders) were then imported into SAS 9.4 for cleaning and data manipulation. Code for preparing the data is available from the corresponding author on reasonable request.
Data analysis	Analysis was done in R version 4.2. SAS datasets were imported using the haven package (version 2.5.0). Multilevel models were fit using the glmmTMB package (version 1.1.3) and model fit was assessed using the DHARMa package (version 0.4.5). Simultaneous multinomial confidence intervals were estimated using the MultinomialCI package (version 1.2). Code for conducting the analysis is available from the corresponding author on reasonable request.

For manuscripts utilizing custom algorithms or software that are central to the research but not yet described in published literature, software must be made available to editors and reviewers. We strongly encourage code deposition in a community repository (e.g. GitHub). See the Nature Portfolio guidelines for submitting code & software for further information.

Data

Policy information about availability of data

All manuscripts must include a data availability statement. This statement should provide the following information, where applicable:

- Accession codes, unique identifiers, or web links for publicly available datasets
- A description of any restrictions on data availability
- For clinical datasets or third party data, please ensure that the statement adheres to our policy

This study used individual patient health data that cannot be shared without ethical approval. Analysis datasets are held at the Australian Institute of Health Innovation and access can be provided to researchers who have received approval from the Sydney Children's Hospital Network Human Research Ethics Committee.

Human research participants

Policy information about studies involving human research participants and Sex and Gender in Research.

Reporting on sex and gender	Patient sex was obtained from the hospital medical records. Sex is not included in the analysis as there was no significant association with any of the study outcomes.
Population characteristics	The study population for the stepped wedge component was all inpatients at a 310-bed paediatric tertiary referral hospital excluding oncology and intensive care units. The 1-year followup included a random sample of patients in the same wards, stratified by ward. Ages ranged from 0 to 18.
Recruitment	This study involved review of medical records. No patient recruitment process was involved.
Ethics oversight	Sydney Children's Hospital Network Human Research Ethics Committee (HREC/15/SCHN/370); Individual patient consent to access retrospective medication and clinical records was waived.

Note that full information on the approval of the study protocol must also be provided in the manuscript.

Field-specific reporting

Please select the one below that is the best fit for your research. If you are not sure, read the appropriate sections before making your selection.

☐ Life sciences ☒ Behavioural & social sciences ☐ Ecological, evolutionary & environmental sciences

For a reference copy of the document with all sections, see nature.com/documents/nr-reporting-summary-flat.pdf

Behavioural & social sciences study design

All studies must disclose on these points even when the disclosure is negative.

Study description	Quantitative stepped wedge cluster randomised controlled trial of electronic medication management, with an additional 1-year post-implementation sample.
Research sample	Stepped wedge period: medication orders for all inpatients at a 310-bed paediatric tertiary referral hospital, excluding oncology and intensive care units. One-year follow-up: medication orders for a random sample of patients in the same wards, stratified by ward. Patient age ranged from 0 to 18 years - median 55.5 months for paper chart period, 85 months for immediate post-eMM period, 80 months for 1 year post-eMM period. 40% were female in the stepped wedge period; 44% were female in the 1 year followup sample.
Sampling strategy	Stepped wedge cluster randomised controlled trial (SWCRCT): all medication orders made in the study wards were reviewed. One year followup: all medication orders for a random sample of patients from the study wards were reviewed.<br>The expected reduction in overall prescribing error rate was 60%, from 4.06 errors per admission (SD=5.27) to 1.62 (SD=2.87) with an estimated intraclass correlation coefficient (ICC) of 0.06. Collecting data on eight clusters would allow detection of a minimum change of 20% for overall errors with 100% power and 42% change for potential ADEs with 83% power (for two-sided tests; alpha<5%). At each step, records for 112 patient admissions were estimated to be required, totalling 1,232 across the study.<br>The 1-year follow-up sample size was calculated based on data from 1817 patients generated during the SWCRCT. Using these data, we calculated a clinical prescribing error rate of 0.24/patient-day (SD=0.78) with an ICC of 0.04 for eight clusters. To detect a 35% change 1 year post-eMM, we required 1230 patient records for the 1-year post-eMM follow-up sample (for a two-sided test; 80% power; alpha<5%).
Data collection	Medication orders were reviewed retrospectively by one of three clinical pharmacists, independent from the hospital, to identify and classify clinical and procedural (stepped wedge period only) prescribing errors. Error data were recorded on paper then entered into a Microsoft Access database for checking. The order in which records were reviewed was randomised across all wards. Errors

	identified by the pharmacists were rated for their potential harm severity using a five-point scale. Any documentation which indicated that staff had detected an error (e.g. note of an order correction; a case conference with parents) was recorded. Details of all medications ordered (date of prescribing, medication name and route) for each patient admission were recorded in Microsoft Excel to provide denominators for error rate calculations.<br>Errors with potential severity of 3-5 and where the medication was administered to the patient, were reviewed by a panel comprising a paediatrician, paediatric nurse and pharmacist to assess if actual harm occurred.<br>Blinding was not possible.
Timing	Stepped wedge: 22 April 2016 to 11 July 2016<br>One year followup: 20 June 2017 to 30 September 2017
Data exclusions	Patients without medication orders were excluded.
Non-participation	Records were reviewed for all patients in the study wards during the study periods.
Randomization	Nine wards were grouped into eight clusters prior to randomisation. Two wards were randomised together as there was direct physical access from one ward to the other, and they treated similar patients. Researchers, blinded to cluster/ward identity and independent of the hospital, randomly assigned (using a computer number generator) the sequence of cluster eMM implementation. The trial had 10 steps of one week duration (ie a 70-day period). The hospital determined the one-week step between cluster implementation.

Reporting for specific materials, systems and methods

We require information from authors about some types of materials, experimental systems and methods used in many studies. Here, indicate whether each material, system or method listed is relevant to your study. If you are not sure if a list item applies to your research, read the appropriate section before selecting a response.

Materials & experimental systems

n/a	Involved in the study
☒	☐ Antibodies
☒	☐ Eukaryotic cell lines
☒	☐ Palaeontology and archaeology
☒	☐ Animals and other organisms
☐	☒ Clinical data
☒	☐ Dual use research of concern

Methods

n/a	Involved in the study
☒	☐ ChIP-seq
☒	☐ Flow cytometry
☒	☐ MRI-based neuroimaging

Clinical data

Policy information about clinical studies

All manuscripts should comply with the ICMJE guidelines for publication of clinical research and a completed CONSORT checklist must be included with all submissions.

Clinical trial registration	Australian New Zealand Clinical Trials Registry (ANZCTR) 370325
Study protocol	Westbrook et al. BMJ Open 2016; 6(10): 1-12. doi: 10.1136/bmjopen-2016
Data collection	Retrospective review of medical records at a paediatric tertiary referral hospital for periods 22 April 2016 to 11 July 2016 and 20 June 2017 to 30 September 2017. Review occurred during 2016-2019.
Outcomes	Primary: clinical prescribing error rates (number of clinical errors per 100 medication orders); rates of potential adverse drug events (potential severity rating 3, 4, or 5).<br>Secondary: error rates by type of error; error rates for high-risk medications; errors where there was documentation that staff had detected the error; errors causing actual harm; procedural prescribing errors.
